# The Gut Microbiota Metabolite Urolithin B Prevents Colorectal Carcinogenesis by Remodeling Microbiota and PD-L1/HLA-B

**DOI:** 10.1155/2023/6480848

**Published:** 2023-02-01

**Authors:** Lixue Wang, Jinlong Chen, Qijun Chen, Hui Song, Ziqian Wang, Wen Xing, Siyao Jin, Xueying Song, Hua Yang, Wenhua Zhao

**Affiliations:** ^1^School of Pharmaceutical Sciences, Capital Medical University, Beijing 100069, China; ^2^School of Basic Medical Sciences, Capital Medical University, Beijing 100069, China; ^3^Clinical Research Center, Beijing Children's Hospital, Capital Medical University, National Center for Children's Health, China 100045; ^4^Central Laboratory, Capital Medical University, Beijing 100069, China

## Abstract

Colorectal cancer has risen to the third occurring cancer in the world. Fluorouracil (5-Fu), oxaliplatin, and cisplatin are the most effective chemotherapeutic agents for clinical chemotherapy. Nevertheless, due to chemotherapeutic drug resistance, the survival rate of patients with CRC remains very low. In this study, we used the inflammation-induced or mutation-family-inherited murine CRC models to study the anticancer and immunotherapy effects of urolithin B (UB), the final metabolite of polyphenols in the gastrointestinal tract. The label-free proteomics analysis and the gene ontology (GO) classifications were used to test and analyze the proteins affected by UB. And 16S rDNA sequencing and flow cytometry were utilized to uncover gut microbiome composition and immune defense improved by UB administration. The results indicated that urolithin B prevents colorectal carcinogenesis by remodeling gut microbial and tumor immune microenvironments, such as HLA-B, NK cells, regulatory T cells, and *γδ* TCR cells, and decreasing the PD-L1. The combination of urolithin B with first-line therapeutic drugs improved the colorectal intestinal hematochezia by shaping gut microbiota, providing a strategy for the treatment of immunotherapy treatment for CRC treatments. UB combined with anti-PD-1 antibody could inhibit the growth of colon cancer. Urolithin B may thus contribute to anticancer treatments and provide a high immune response microenvironment for CRC patients' further immunotherapy.

## 1. Introduction

Colorectal cancer (CRC) is the third most prominent cause of cancer-associated mortality worldwide [1]. The World Health Organization reported that there were 1.93 million new cases of cancer in 2020. Colorectal cancer has risen to the third occurring cancer in the world. In 2020, the incidence of colorectal cancer, with 550 thousand, ranked second in China [2]. The prognosis of colorectal cancer is related to its clinical stage. According to the American Joint Committee on Cancer staging system, the five-year survival rate is less than 60% for patients with colorectal cancer and lymph node metastasis (stage III) and less than 10% for patients with distal metastasis (stage IV) [3]. Cytotoxic chemotherapy and radio-chemotherapy are used to treat patients with stage III CRC [4]. Fluorouracil (5-Fu), oxaliplatin, and cisplatin are the most effective chemotherapeutic agents for clinical chemotherapy [5, 6]. Nevertheless, due to chemotherapeutic drug resistance, the survival rate of patients with CRC remains very low [7–9]. Early discontinuation is common in stage III colon cancer patients receiving chemotherapy [10]. The discovery of anti-colon cancer drugs has become an urgent problem to be solved.

Notably, in the tumor microenvironment of colorectal cancer, the increased proportion of regulatory T (Treg) cells of colorectal patients is the key factor of tumor immune escape [11, 12]. Besides, the number of natural killer cells (NK) is positively correlated with the clinical prognosis of colorectal patients [13, 14]. What is more, the natural immune cell, *γδ* T cell, is mainly distributed in the intestinal epithelium and has a protective effect on the intestine [15, 16]. In addition, gut microbiota also plays an important role in the colon cancer microenvironment. For example, *Alloprevotella* is the pathogenic bacteria in CRC development [17, 18], and *Akkermansia muciniphila* [19–22] is the immunomodulatory bacteria in cancer development. *Akkermansia muciniphila* was related to the favorable therapeutic efficacy of immunotherapy, and Bacteroides has been considered an unfavorable bacteria [23, 24].

Dietary supplements, such as polyphenols, provide multiple favorable benefits, including anticarcinogenic benefits [25, 26]. Urolithins, which are dibenzopyran-6-one derivatives, are key metabolites of polyphenols in the gastrointestinal (GI) tract. Urolithins have anti-inflammatory [27–30], cardiovascular-protective [31–34], and antimicrobial [35] effects in vitro. Previous studies have revealed that urolithins have anticarcinogenic effects on castration-resistant prostate cancer [36], hepatocellular carcinoma [37], endometrial carcinoma [38], melanoma [39], breast cancer [40, 41], pancreatic cancer [42], bladder cancer [43], and colon cancer [44]. Urolithin B, which is the final metabolite of polyphenols in the GI tract [45], transformed from the intestinal metabolites of pomegranate ellagitannins by high-speed countercurrent chromatography [46]. Notably, whether urolithins suppress the progression of colorectal carcinogenesis by influencing gut microbiota or tumor immune microenvironment remains unknown.

Therefore, in this study, we investigated the cancer-preventive properties of urolithin B on colorectal carcinogenesis. The regulations of gut microbiota, tumor immune microenvironment, and PD-L1 were also investigated.

## 2. Materials and Methods

### 2.1. Animal

Male C57BL/6 mice, 8 weeks, weighing 18-20 g, purchased from Shanghai Model Organisms Center (Inc) were used, maintained in Chinese Experimental Animal Resources Research Institute for food and drug control (SPF standards, room temperature at 20-25°C, relative humidity 50%, 12 h light/12 h dark cycle). All groups were fed with a regular diet of standard feedstuff freely. The studies were approved by the Experimental Animal Management Committee at Capital Medical University (AEEI-2021-254).

Apc^min/+^ mice with a C57BL/6 background were obtained from Shanghai Model Organisms Center (Inc) and maintained in a standard environment in Chinese Experimental Animal Resources Research Institute for food and drug control. The progeny of Apc^min/+^ intercrosses were genotyped by PCR analysis with DNA isolated from the tail with the Genomic DNA Mini Preparation Kit with Spin Column (D0063, Beyotime) using the following two primers: 5′-GTGCAGCAGCTTTAAGGAA-3′ and 5′-AATGGAACTCGGTGGTAGA-3′. The DNA sequencing was performed with the former primer: GACATGATGATAGTAGGTCAGACAATTTCAATACTGGAAACATGACTGTTCTTTCACCATATTTAAATACTACGGTATTGCCCAGCTCTTCTTCCTCAAGGGGAAGTTTAGACAGTTCTCGTTCTGAGAAAGACAGAAGTT (T/A) GGAGAGAGAGCGAGGTATTGGCCTCAGTGCTTACCATCCAACAACAGAAAATGCAGGAACCTCATCAAAACGAGGTCTGCAGATCACTACCACTGCAGCCCAGATAGCCAAAGTTATGGAAGAAGTATCAGCCATTCATACCTCCCAGGACGACA (wild type-T and mutant-A).

### 2.2. CRC Model of Colorectal Tumorigenesis and Treatment

Male C57BL/6 mice were used at the age of 8 weeks, administrated with 12.5 mg/kg AOM (A5486, Sigma, i.p.). One week later, 2.5% DSS (MB5535, Meilunbio) was given in drinking water for one week and then regular drinking water for two weeks. The DSS cycle was repeated three times, and the experiment lasted for 70 days.

APC^min/+^ mice were used at the age of 8 weeks, with 2.5% DSS (MB5535, Meilunbio) given in the drinking water for one week followed by regular drinking water for two weeks. The experiment cycle was repeated twice and lasted for 50 days.

Treatment groups of low, middle, and high or positive control were given a gavage of 10 mg/kg, 20 mg/kg, and 40 mg/kg urolithin B or 30 mg/kg capecitabine dissolved in 0.25% CMC-Na, and the combination group was given both 30 mg/kg capecitabine and 20 mg/kg UB, while the model group was given the same volume of distilled water, with no administration to the control group. For adjuvant immunotherapy, the mice were administrated with 100 *μ*g anti-PD-1 (clone RMP1-14, BioXcell, i.p.) once a week with or without 20 mg/kg UB.

All mice were weighed regularly. In the end, collect the colorectum of the mice after sacrifice and wash with PBS, to calculate tumor load and prepare them for further experiments.

Tumor load was calculated according to the diameter of the tumors. The tumor load of each mouse is equal to the sum of the average diameters of all tumors on its large intestine.

### 2.3. Label-Free Proteomics Analysis

SW480 cells were treated with 0.15 or 15 *μ*M urolithin B. Next, cells were lysed, and the concentration of extracted protein was tested by the BCA protein assay kit (Beyotime, P0012). Lyophilizate of the proteins was redissolved, centrifuged, and isolated through the C_18_ column, tested by LC-MS/MS, followed by STRING network analysis showing proteins regulated by UB.

### 2.4. Gut Microbiota Analysis for AOM/DSS CRC Mice

Feces of the mice were collected in the last few days of the test for 0.2-0.4 g/tube (about 3 grains) and stored at -80°C for further detection. Gut microbiota was analyzed by 16S rDNA sequencing, and the data were analyzed on the online platform of the Majorbio Cloud Platform (Shanghai Majorbio Bio-pharm Technology Co., Ltd). The operational taxonomic units (OUT) were used for alpha diversity analysis and beta diversity analysis and linear discriminant analysis (LDA).

### 2.5. Flow Cytometry Analysis of the CRC and Spleen Tissue

The colon and rectum with adenoid tumor were collected and digested with the Lamina Propria Dissociation Kit, mouse (130-097-410), according to the manufacturer's direction.

The spleens were ground into single cells and lysed with red blood cell lysis buffer (R1010, Solarbio) on ice after centrifugation; the followed steps were done according to the instruction. All the procedures were carried out according to BD procedure steps with anti-mouse antibody for CD45 (103111/103137, BioLegend), CD3 (100203, BioLegend), CD4 (100407, BioLegend), CD8a (100709, BioLegend), TCR*γ*/*δ* (118129, BioLegend), NK1.1 (108713, BioLegend), F4/80 (123107, BioLegend), FOXP3 (126419, BioLegend), CD25 (101917, BioLegend), granzyme B (372216, BioLegend), CD16/32 (101319, BioLegend), CD11b (101208, BioLegend), CD11c (1173229, BioLegend), Ly-6C (128017, BioLegend), and Ly-6G (127613, BioLegend). After being blocked with TruStain FcX (anti-mouse CD16/32) antibody, cells were stained with antibodies for cell surface markers and then the intracellular biomarker. True-Nuclear™ Transcription Factor Buffer Set (424401, BioLegend) and fixation buffer (BioLegend, 420801) were used according to the protocol recommended by BioLegend. Before that, BD Compensation Beads (BD Biosciences, 552845) were used to make the best fluorescence compensation settings for multicolor flow cytometric analysis.

### 2.6. Immunohistochemistry

Mouse CRC tissues were embedded in paraffin and made into slicers. Then, the slicers were deparaffinized in xylene and hydrated into water with graded ethanol (5 minutes in 100%, 5 minutes in 100%, and 5 minutes in 95%, then 5 minutes in 75%).

For HE or PCNA staining, procedures were performed according to instructions (G1005, Servicer Bio) provided by the manufacturer. For the immunohistochemical experiment, slicers were pretreated with antigen retrieval with citrate (G1202-250ML) or Tris-EDTA (G1203-250ML) and then incubated with PBST (PBS containing 0.3% Triton X-100) for 20 minutes and incubated with 3% H_2_O_2_ in methanol at 37°C for 20 minutes. After being blocked with goat serum (BOSTER, AR1009) in PBST for 1 h, tissue slides were incubated at 4°C overnight with primary antibodies as follows: anti-PD-L1/CD274 antibody (K009918P, Solarbio). The sections were then washed with PBST for 3 times and incubated with goat anti-rabbit IgG antibody (HRP) (ARG65351, Arigo) at 25°C for 30 minutes. Then, the slicers were treated with the DAB Substrate Kit (20x) (Servicebio, G1212-200T) and then counterstained with hematoxylin. All the slides were imaged at the end for 40x amplification.

### 2.7. Western Blot Analysis

Colon tissues were isolated and dissected from mice, homogenized, and lysed with ice-cold RIPA Lysis Buffer (P0013C, Beyotime) by a high-speed low-temperature tissue grinding machine, then centrifuged at 8000g for 10 min at 4°C. The protein concentrations were measured using the BCA protein assay kit (Beyotime) according to the instructions. Then, the total proteins (50 *μ*g) were separated with 10% SDS-PAGE and transferred onto a PVDF membrane (Millipore). The membrane was then blocked with 5% skimmed milk powder (P1622, Applygen) dissolved in 1x PBST (RBU164-500, Beijing Roby Biotechnology) for 1 h at room temperature and incubated with primary antibodies overnight at 4°C. The prime antibodies including anti-HLA-B antibody (K009916P, Solarbio) was diluted in blocking solution (1 : 1000, P0252-100ml, Beyotime). And *β*-actin (K006153P, Solarbio) was used as an internal control. After that, HRP-labeled goat anti-rabbit IgG (L3012, SAB) was used as the secondary antibody to be incubated with the membrane. Finally, the Immobilon Western HRP Substrate (WBKLS0500, Millipore) was used for the chemiluminescence detection of the bounded antibody.

### 2.8. Statistical Analysis

All experiments were performed at least three times. The data are presented as the mean ± SE. Statistical differences between the control group and treated groups were evaluated using Student's *t*-test or the Student-Newman-Keuls multiple comparison test. Differences between groups are considered statistically significant at *p* < 0.05.

## 3. Results and Discussion

### 3.1. The Effect of Urolithin B on AOM/DSS and Apc^min/+^ Colorectal Carcinogenesis

**Figure 1:**
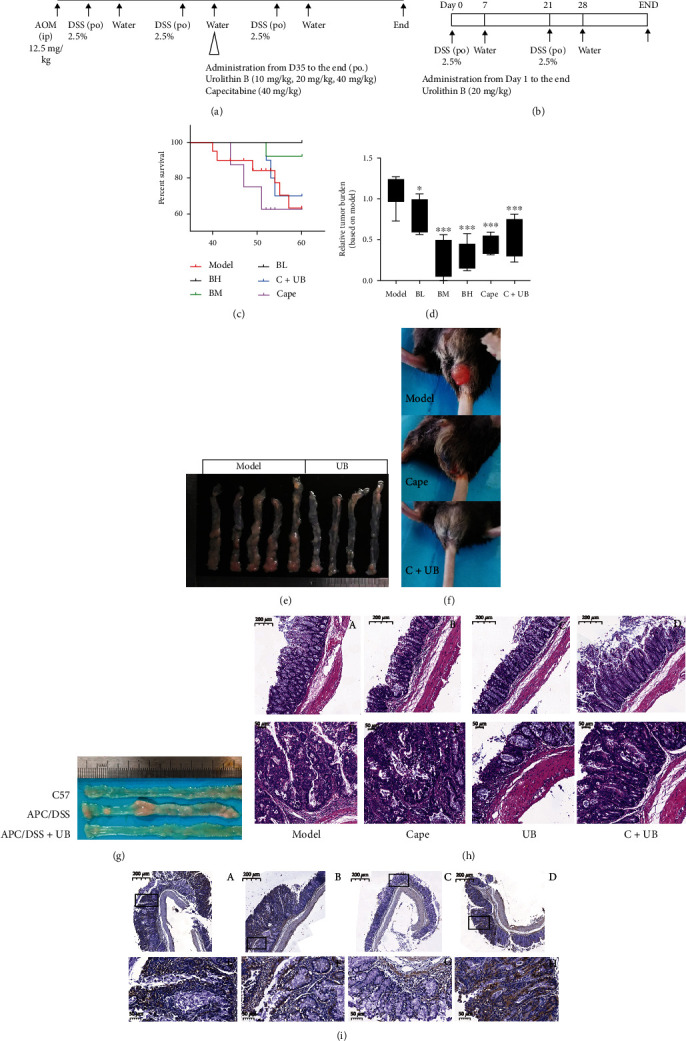
Urolithin B decreased tumor growth both in the AOM/DSS model and in the APC^min/+^ mice. Experimental design of AOM/DSS (a) or APC^min/+^ (b) tumorigenesis, indicating the time and dose of administration in different groups. (c) Survival rate of different groups in AOM/DSS-induced CRC mice shows that UB could improve physical condition and prolong living time in AOM/DSS mice. (d) The tumor size of different groups of AOM/DSS mice after therapy with urolithin B or/and capecitabine, calculated on the sum of average diameter of all polyps on a colon in each group. Data are shown as mean ± SD. (e) Macroscopic scope of colorectum from the mice shows that the administration of urolithin B reduced the tumorigenesis in AOM/DSS-induced CRC mice. (f) The intestinal bleeding induced by DSS irritation. (g) Typical macroscopic scope of colorectum from APC (adenomatosis polyposis coli) mutation model mice with/without treatment of UB. (h) HE staining of different groups in the AOM/DSS model. A–D are peritumoral positions, and E–H are typical adenomatous polyps. (i) Immunohistochemical staining in all groups of AOM/DSS mice treated with antibody against PCNA shows that urolithin B restrained the expression of PCNA on AOM/DSS mice. (h, i) The same group division (A, E: M; B, F: C; C, G: UB; D, H: CB) and magnification bars for A–D 200 *μ*m and E–H 50 *μ*m. M: AOM/DSS colorectal cancer group; UB: M group administrated with 20 mg/kg urolithin B (BH-40 mg/kg, BM-20 mg/kg, and BL-10 mg/kg); C: M group administrated with 30 mg/kg capecitabine; C+UB: M group administrated with both urolithin B and capecitabine. ^∗^*p* < 0.05,  ^∗∗^*p* < 0.01, and^∗∗∗^*p* < 0.001, compared to the AOM/DSS colorectal cancer group.

In both the AOM/DSS colorectal carcinogenesis (CRC) model (Figure 1(a)) and the Apc^min/+^ CRC model (Figure 1(b)), the fur of mice was disordered and their food intake was reduced after DSS treatment. Visible bloody stool began to appear on the fifth day after drinking DSS. Some of the mice in the AOM/DSS model died after drinking DSS water during the second and third cycles (Figure 1(c)). Compared with the CRC model, the administration of 20 mg/kg UB improved the survival rate; however, the administration of capecitabine failed to improve the survival rate (Figure 1(c)). In addition, the administration of UB reduced the tumor size in both AOM/DSS (Figures 1(d) and 1(e)) and Apc^min/+^ CRC (Figure 1(g)) models. Compared with the AOM/DSS model, administration of UB (*p* < 0.01) or capecitabine (*p* < 0.001) could reduce the adenoma burden of AOM/DSS CRC mice (Figure 1(d)). Although the UB combined with capecitabine group shows no changes in the tumor load and survival rate compared to the capecitabine group (Figures 1(c) and 1(d)), the UB combined with capecitabine group has less intestinal hematochezia (Figure 1(f)).

The colons with adenoma from AOM/DSS mice were sliced and stained with hematoxylin and eosin (Figure 1(h)) or stained by immunohistochemistry with anti-PCNA antibody (Figure 1(i)). Compared with the c57 group, the colons of the AOM/DSS model lost the goblet cells, crypts, and epithelial cells and formatted the atypical hyperplasia or tubular adenoma or adenocarcinoma. The administration of UB fixed those disorders and reduced the expression of PCNA on colons of CRC mice compared with AOM/DSS model mice (Figure 1(i), B). Additionally, there were intestinal pathological changes such as intestinal obstruction, flatulence, severe intestinal bleeding, and anal prolapse during the AOM/DSS modeling process. Compared with the AOM/DSS or Apc^min/+^ CRC model group, urolithin B could obviously reduce the tumor load and effectively inhibit colorectal carcinogenesis.

### 3.2. Label-Free Proteomics Analysis of Urolithin B-Regulated Proteins

**Figure 2:**
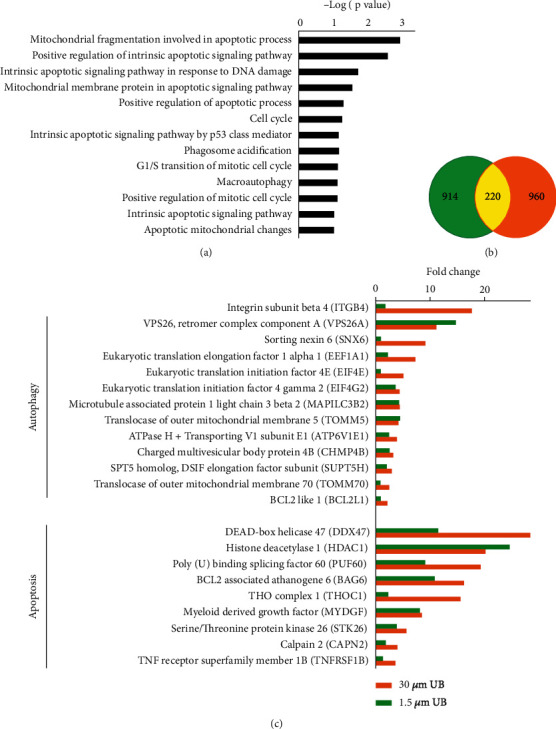
A histogram showing the gene ontology (GO) classifications of SW480 cells treated with urolithin B determined by label-free proteomics analysis. (a) Significantly (Benjamini-Hochberg FDR < 0.05) overrepresented GO-BPs in hyperactivated proteins after urolithin B treatment are shown on a bar graph. (b) A summary of coregulated proteins between both groups. Green: 0.15 *μ*M UB-treated group; orange: 15 *μ*M UB-treated group. (c) Differentially expressed proteins that are closely related to apoptosis and autophagy functions in the 0.15 and 15 *μ*M UB-treated groups are shown.

**Figure 3:**
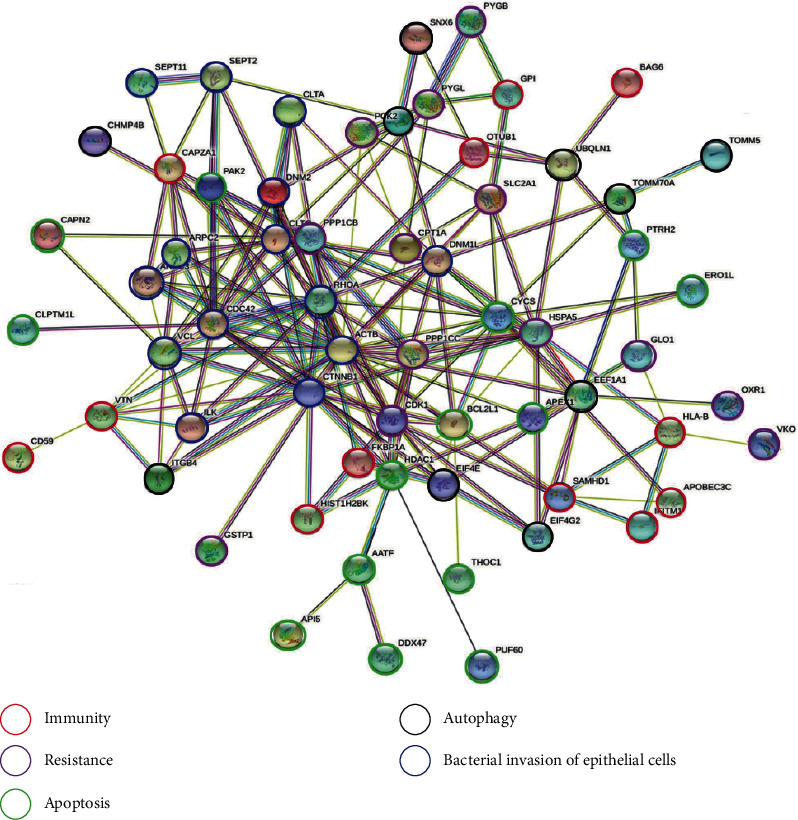
STRING network analysis showing the direct roles of UB on related immunity/drug resistance/autophagic/apoptotic/bacterial invasion-related pathways. Immunity-related proteins (red circles), resistance-related proteins (purple circles), apoptosis-related proteins (green circles), autophagy-related proteins (black circles), and bacterial invasion of epithelial cells-related proteins (blue circles) are shown.

Next, the protein expression levels of SW480 cells treated with urolithin B were analyzed by proteomics analysis. Protein abundance ratios > 2.0 and <0.5 combined with a *t*-test (*p* < 0.05) between samples from two groups were used to identify differentially expressed proteins. A total of 914 and 960 upregulated proteins in response to 0.15 and 15 *μ*M UB treatments, respectively, were identified by label-free proteomics (Figures 2(a) and 2(b)). The biological process of UB treatment showed that UB upregulated mitochondrial fragmentation that is involved in the apoptotic process, the positive regulation of the intrinsic apoptotic signalling pathway, the intrinsic apoptotic signalling pathway in response to DNA damage, mitochondrial membrane proteins in the apoptotic signalling pathway, the positive regulation of the apoptotic process, the cell cycle, the intrinsic apoptotic signalling pathway by a p53 class mediator, phagosome acidification, G1/S phase transition of the mitotic cell cycle, and apoptotic mitochondrial changes (Figure 2(a)). Autophagy-upregulated proteins with fold changes greater than 2 and apoptosis-upregulated proteins with fold changes greater than 3.5 are shown in Figure 2(c). STRING network analysis showed that UB-regulated immunity-related proteins (Figure 3, red circles), UB-regulated resistance-related proteins (Figure 3, purple circles), UB-regulated apoptosis-related proteins (Figure 3, green circles), UB-regulated autophagy-related proteins (Figure 3, black circles) and UB-regulated bacterial invasion of epithelial cells-related proteins (Figure 3, blue circles) interacted with each other. In general, UB was shown to regulate immunity-related proteins, autophagy-related proteins, apoptosis-related proteins, resistance-related proteins, and bacterial invasion of epithelial cells-related proteins in colon cancer cells.

### 3.3. Urolithin B Regulated Gut Microbiota in AOM/DSS-Induced Colorectal Cancer (CRC) Mice

**Figure 4:**
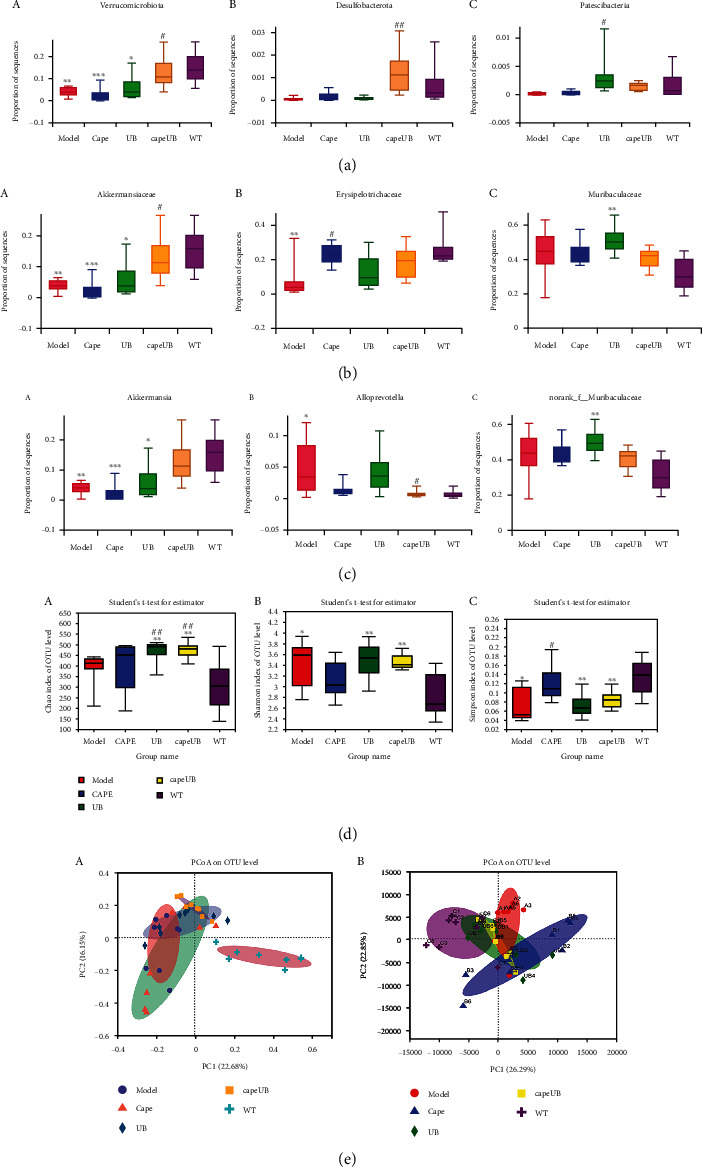
Alpha and beta diversity analysis and significant difference test of the gut microbiota in all groups. Bar plots of relative abundance on phylum level (a), family level (b), and genus level (c) including Verrucomicrobiota (a, A), Desulfobacterota (a, B), and Patescibacteria on phylum level (a, C); Akkermansiaceae (b, A), Erysipelotrichaceae (b, B), and Muribaculaceae (b, C) on family level; and Akkermansia (c, A), Alloprevotella (c, B), and norank_f_Muribaculacea (c, C) on genus level. (d) UB could improve the community richness in AOM/DSS-induced CRC mice. Community richness and diversity in all groups were shown in Chao (A), Shannon (B), and Simpson (C) indices. (e) PCA (Principal Component Analysis) using Euclidean distance (B) and PCoA (principal coordinate analysis) calculated by Bray-Curtis (A) both at the OTU level separating groups from each other. UB: AOM/DSS model mice treated with 20 mg/kg urolithin B; CAPEUB: treatment group treated with capecitabine and 20 mg/kg urolithin B. ^∗^*p* < 0.05 vs. WT group, ^∗∗^*p* < 0.01 vs. WT group, and ^∗∗∗^*p* < 0.001 vs. WT group; ^#^*p* < 0.05 vs. model group, ^##^*p* < 0.01 vs. model group. Error bar indicates SD; *n* = 8 for each group.

**Figure 5:**
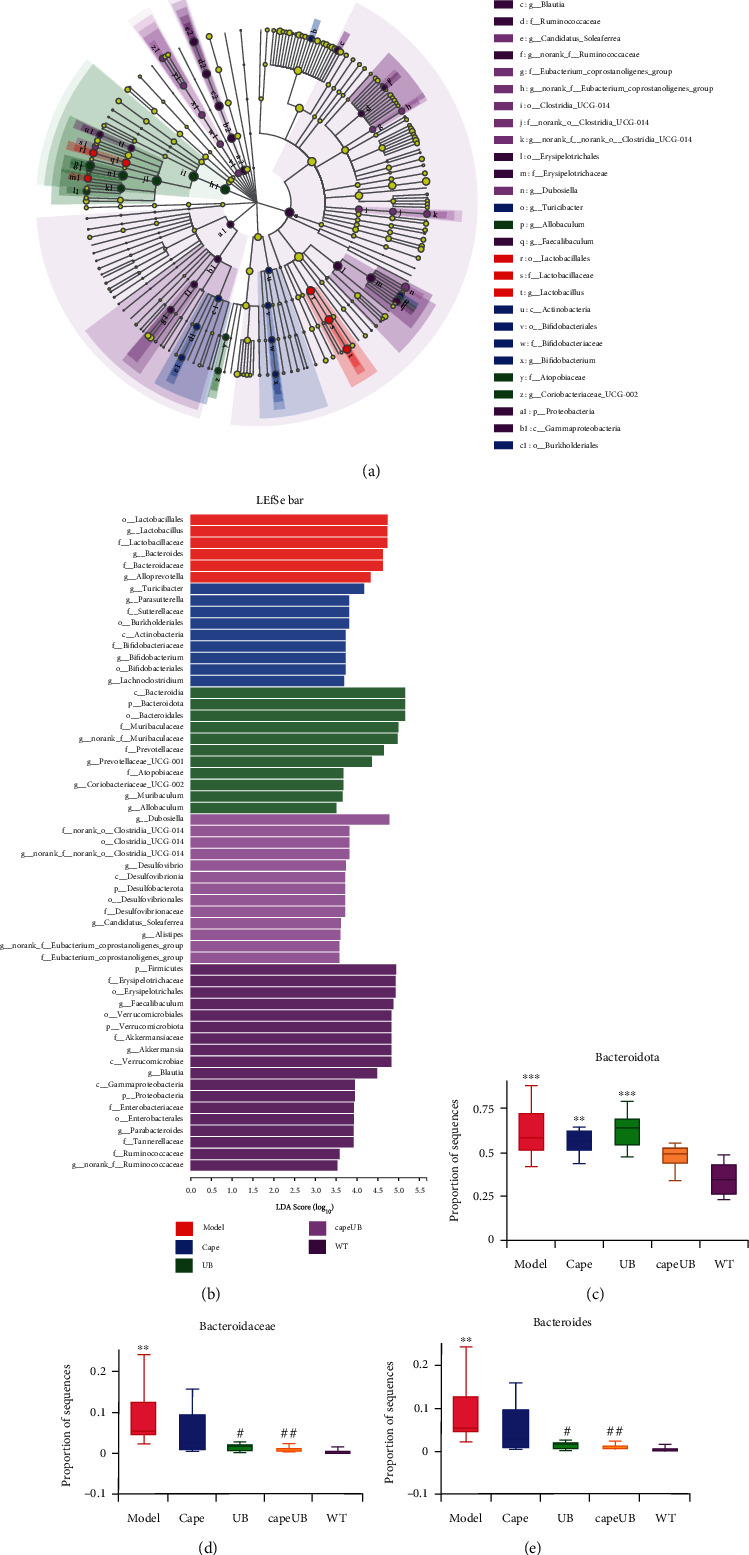
Urolithin B changed AOM/DSS colorectal cancer-induced gut microbiota dysbiosis in mice. Taxa between different groups analyzed by LEfSe were shown in cladogram (a) and histogram (b) (Kruskal-Wallis test, LDA > 3.5 and *p* value < 0.05). Bar plots of relative abundance on phylum level of Bacteroidota (c), family level of Bacteroidaceae (d), and genus level of Bacteroides (e) in different groups were shown here. ^∗^*p* < 0.05 vs. WT group, ^∗∗^*p* < 0.01 vs. WT group, and ^∗∗∗^*p* < 0.001 vs. WT group; ^#^*p* < 0.05 vs. model group, ^##^*p* < 0.01 vs. model group. Error bar indicates SD; *n* = 8 for each group.

A comparison of gut microbial diversity indexes was carried out by 16S rDNA sequencing and bioinformatic diversity analysis.  Figure 4 shows the Shannon and Simpson indexes of alpha diversity results concerning the microbia in the AOM/DSS colon compartment with or without urolithin B supplementation. Compared to capecitabine, the first-line therapeutic drug group, the urolithin B group significantly increased the Shannon index of intestinal flora (*p* < 0.05, Figure 4(d), B). The Chao index of the microbiota of the urolithin B group was higher than that of the AOM/DSS group (*p* < 0.05, Figure 4(d), A). After the intervention of capecitabine combined with urolithin B, the microbial structure deviated from the CRC group and approached the c57 group (Figure 4(e)). As shown in Figures 4(a)–4(c) and 5(c)–5(e), compared to the AOM/DSS CRC group, urolithin B decreased the Bacteroides at the family (*p* < 0.05) and genus (*p* < 0.05) levels; after the combination of capecitabine and urolithin B, the abundance of Verrucomicrobiota (*p* < 0.05) and Desulfobacterota (*p* < 0.01) was increased on the phylum level, the abundance of Akkermansiaceae (*p* < 0.05) was increased at the family level, and the abundance of *Alloprevotella* was decreased significantly (*p* < 0.05) at the genus level.

LEfSe results (Figures 5(a) and 5(b)) revealed that urolithin B and capecitabine significantly changed the constitution of the gut microbiota in AOM/DSS CRC mice. The dominant flora of the urolithin B group is c_Bacteroidia, p_Bacteroidota, o_Bacteroidales, and f_Muribaculaceae; that of the capecitabine combined with urolithin B group is *g_Dubosiella*, f_norank_o_Clostridia UCG-014, o_Clostridia UCG-014, *g_norank_f_orank_o_Clostridia UCG-014*, and *g_Desulfovibrio*. Compared with the AOM/DSS group, UB decreased the abundance of Bacteroidaceae and Bacteroides, and so did the UB combined with capecitabine group (Figures 5(c)–5(e)).

In summary, urolithin B increased the favorable immunotherapy bacteria, *Akkermansia muciniphila*, and decreased the unfavorable immunotherapy bacteria, *Bacteroides* and *Alloprevotella*, in AOM/DSS CRC mice.

### 3.4. Urolithin B Regulated Immune Factors in Colorectal Tumor Immune Microenvironment of AOM/DSS-Induced Colorectal Cancer (CRC) Mice

Natural killer cells (NK cells) are one of the primary cells of the innate immune system. NK cells do not need to bind to related antigens and can directly kill tumor cells with missing protomolecules. *γδ* T cells are innate immune cells. The direct tumor-killing effect of *γδ* T cells is a non-MHC restricted tumoricidal effect. The number of NK cells and *γδ* T cells in the colon tumor microenvironment of AOM/DSS colorectal cancer mice treated with or without urolithin B (20 mg/kg) was analyzed by flow cytometry.

**Figure 6:**
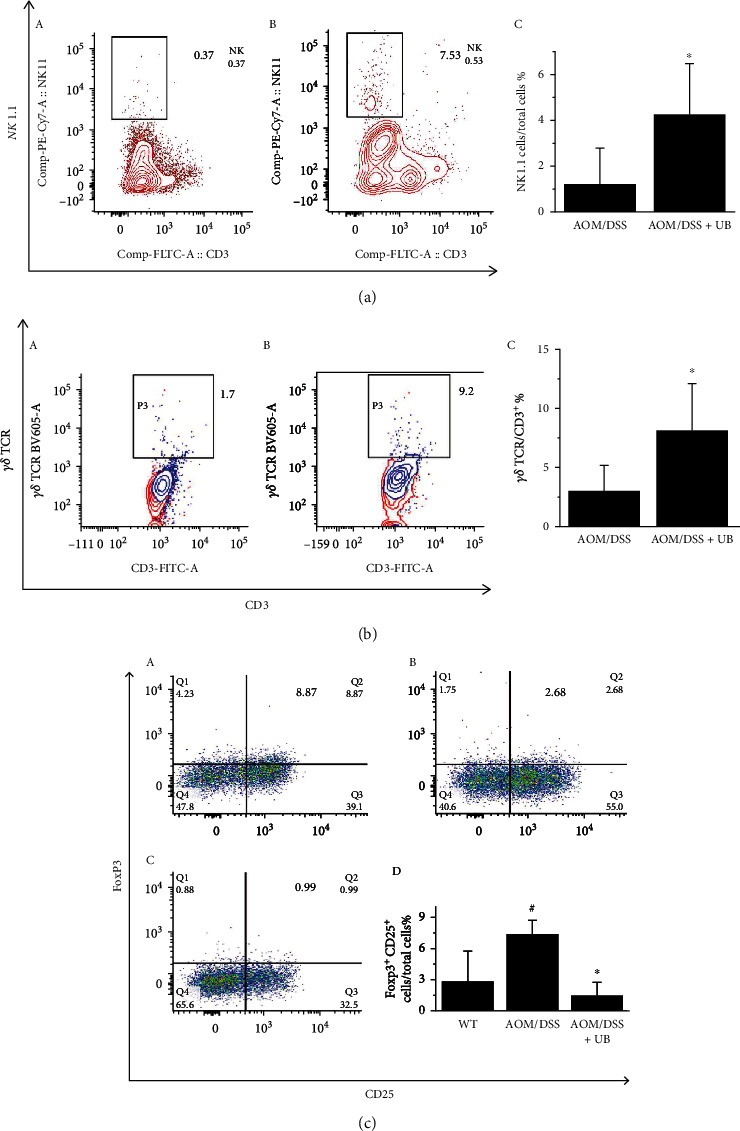
Administration of urolithin B (UB) regulated immune factor in colorectal tumor microenvironment of AOM/DSS-induced colorectal cancer (CRC) mice. (a) Administration of urolithin B (UB) increased percentage of natural killer cells (NK cells). Flow cytometry result of NK ratio in the AOM/DSS colorectal cancer group (AOM/DSS) (A) and treatment group (B) with 20 mg/kg urolithin B (AOM/DSS+UB) once a day after 35 days from the beginning. Statistical analysis of NK profile in tumor microenvironment (C). (b) Administration of urolithin B (UB) increased percentage of *γδ* T cells. Flow cytometry result of *γδ* T cell ratio in group of AOM/DSS colorectal cancer mice (AOM/DSS) (A) and treatment group (B) treated with 20 mg/kg UB once a day after 35 days from the beginning. Statistical analysis of *γδ* T cell profile in tumor microenvironment (C). (c) Administration of urolithin B (UB) reduced percentage of regulatory T cells (Treg). Flow cytometry result of regulatory T cell (Treg) ratio in spleen of AOM/DSS colorectal cancer mouse (AOM/DSS) group (A) and treatment group (B) treated with 20 mg/kg urolithin B (AOM/DSS+UB) once a day after 35 days from the beginning, the control (c57) group (C). Statistic flow analysis of Treg profile in spleen (D). ^∗^*p* < 0.05 compared with AOM/DSS colorectal cancer group. ^#^*p* < 0.05 compared with c57 group. *n* ≥ 6.

Compared with the AOM/DSS group, the number of NK cells in the oral administration group of urolithin B was significantly increased from 1.21 ± 1.56 to 4.25 ± 2.22 (*p* < 0.05, Figure 6(a)); *γδ* TCR expression was significantly increased from 2.98 ± 2.14 to 8.05 + 3.94 (*p* < 0.05, Figure 6(b)). Regulatory T cells (Treg) are immunosuppressive cells in tumors; CD4^+^ CD25^+^ Foxp3^+^ is the specific marker. The spleen marker molecule CD4^+^ CD25^+^ Foxp3^+^ of animals with colon cancer was analyzed by flow cytometry (Figure 6(c)). The proportion of Treg cells was induced in the progression of colon cancer development from 2.83 ± 2.93 to 7.4 ± 1.32 (*p* < 0.05, Figure 6(c)), and the oral administration of urolithin B significantly reduced the percentage of Treg cells in the spleen of colon cancer animals from 7.4 ± 1.32 to 1.49 ± 1.24 (*p* < 0.05, Figure 6(c)). The results showed that urolithin B significantly increased the number of NK cells and *γδ* T cells in the tumor microenvironment of colon cancer, inhibited the number of Treg cells, and finally played an anti-colon cancer role.

**Figure 7:**
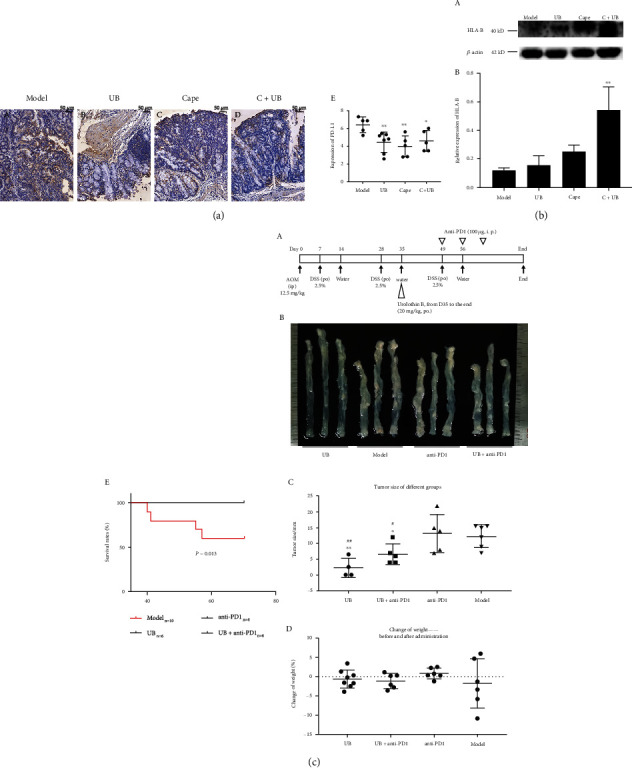
UB combined with anti-PD-1 antibody could inhibit the growth of colon cancer. (a) Immunohistochemistry of PD-L1. (A) AOM/DSS CRC model. (B) CRC administrated with UB (UB). (C) CRC administrated with capecitabine (C). (D) CRC administrated with both UB and capecitabine (C+UB). (E) Calculation of the PD-L1 expression in different groups. (b) The expression of HLA-B. (A) The combination of UB and capecitabine increased the expression of HLA-B. (B) Statistic analysis of WB results. (c) UB combined with anti-PD-1 antibody could inhibit the growth of colon cancer. (A) Schematic of UB and/or anti-PD-1 treatment. (B) Representative samples of UB, model, anti-PD-1, UB, and anti-PD-1 treatment. (C) Statistic analysis of tumor size in different groups (sum of mean tumor diameter of every mice). (D) Weight change during the treatment of four groups (day 35 to the end). (E) Survival rate during the treatment. Mean ± SD, one-way ANOVA, ^∗^*p* < 0.05,  ^∗∗^*p* < 0.01, and^∗∗∗^*p* < 0.001, compared with AOM/DSS colorectal cancer group, *n* ≥ 6.

Notably, UB inhibits colon cancer and regulates the immune response of colon cancer. UB inhibited the expression of PD-L1 (Figure 7(a)). A combination of UB and capecitabine increased the expression of HLA-B (Figure 7(b)). In the experiment of UB combined with anti-PD-1 antibody (Figure 7(c), A), compared to the AOM/DSS colonic cancer model group, UB and UB combined with PD-1 antibody significantly prolonged the survival time of experimental animals (Figure 7(c), E). UB and UB combined with anti-PD-1 antibody significantly reduced the tumor load (Figure 7(c), B and C) but did not affect the weight change of experimental animals (Figure 7(c), D). UB combined with PD-1 antibody shows better effect on the tumor load reduction than the PD-1 antibody group (Figure 7(c), D). The results showed that UB combined with anti-PD-1 antibody could inhibit the growth of colon cancer and be beneficial to the immunotherapy of colon cancer.

## 4. Discussion

Generally, UB prevented colorectal carcinogenesis by shaping gut microbial and tumor immune microenvironment, enhancing the vitality of NK cells, inhibiting the activity of regulatory T cells and the expression of PD-L1, and upregulating the expression of HLA and *γδ* TCR. UB combined with anti-PD-1 antibody could inhibit the growth of colon cancer and be beneficial to the immunotherapy of colon cancer. The combination of UB with first-line therapeutic drugs further improved the anticancer therapy effect and was favorable to the colon cancer immune microenvironment by regulating the composition of immunomodulatory bacteria.

Inverse correlations between the consumption of plant-based foods and mortality as a result of chronic diseases, such as cardiovascular diseases, neurodegenerative diseases, and cancer, have been investigated. Urolithin B, the final metabolite of polyphenols produced by gut microbiota through the loss of one of the two lactones, is a dibenzopyran-6-one derivative [45, 46]. Based on *in vitro* studies, the health benefits attributed to urolithins are numerous and diverse, from antimalarial properties and topoisomerase inhibition to quenching of bacterial quorum sensing. *In vivo* studies conducted in colon cancer are very relevant since the colon is the portion of the GI tract where urolithins are produced and achieve bioactive concentrations.

As an adjuvant therapy, FOLFOX chemotherapy can improve the postoperative survival rate of patients with stage III/II colon cancer patients [4–6]. However, adjuvant chemotherapy leads to the incidence of bone marrow suppression, and some patients fail to complete the established chemotherapy regimen [10]. In recent years, immunotherapy has developed into an effective strategy for treating advanced cancer [47, 48]. However, after surgery, the effect of immunotherapy will be affected by the reduction of the immune response [3]. Therefore, improving the tumor immune microenvironment of CRC patients has a very important guiding significance for clinical treatment.

The regulatory T cells (Tregs) are immunosuppressive in the tumor microenvironment of colon cancer. UB is a high content of multiple intestinal metabolites in food, natural drugs, and traditional Chinese medicine. It is produced by intestinal metabolism in the gastrointestinal tract. This study shows that UB can regulate the tumor microenvironment of colon cancer, upregulate NK cells and *γδ* T cells, inhibit Treg cells, improve immune response, PD-L1, and upregulate the response of immune regulatory flora after combination with capecitabine. Capecitabine is the first-line drug for chemotherapy treatment; after chemotherapy failure, colon cancer patients will further consider improving immune response in immunotherapy. Reducing the immunosuppressive tumor microenvironment and building a high immune response platform will play an important role in the efficient immunotherapy treatment of colon cancer patients. In the present study, the application of UB may provide a high immune response environment for CRC patients' further immunotherapy.

Intestinal flora in the tumor microenvironment of colon cancer is related to immune response. The immunogenicity of colon cancer is weak, and the pathogenic intestinal bacteria increase after colon cancer surgery and chemotherapy [19–24]. The immune response of intestinal flora plays a regulatory role in immunotherapy. Studies have shown that *Akkermansia muciniphila* is immunomodulatory bacteria [19–22]. *Akkermansia muciniphila* was related to the favorable therapeutic efficacy of immunotherapy, and *Bacteroides* has been considered unfavorable bacteria. The high abundance of those immunomodulatory bacteria in the intestine can upregulate the immune response and improve the treatment rate. *Akkermansia muciniphila* (*A. muciniphila*), one of the members of immunomodulatory bacteria, is an anaerobic gram-negative strain that colonizes the mucus layer of mucin-rich ileum and colon. *A. muciniphila* with appropriate abundance provides nutritional support for intestinal epithelial cells, which can protect an intestinal mucosal barrier, enhance intestinal epithelial integrity, reduce inflammatory response, improve glucose and lipid metabolism, and participate in immune response [49–51]. The destruction of microbial structure and the loss of specific bacteria interfere with the efficacy of immunotherapy. By comparing the intestinal flora of responders and nonresponders, the researchers found that the relative abundance of *A. muciniphila* in tumor patients was correlated with the immune response, and the abundance of *A. muciniphila* increased in patients who responded to immunotherapy [19–22]. It is worth noting that *A. muciniphila* can enhance the immune response to chemotherapeutic drugs [21, 22]. Routy et al. summarized the positive correlation between *A. muciniphila* preclinical tumor model and anticancer immune response in cancer patients [19]. In the present study, we showed that UB significantly upregulated *A. muciniphila* level and downregulated *Bacteroidales* and *Alloprevotella*. So, in the primary site of intestinal metabolism, urolithin may upregulate the abundance of immunomodulatory bacteria and further regulate the immune response. Upregulating the abundance of immunomodulatory bacteria and thus regulating the immune response may be one of the mechanisms of urolithin's anti-colon cancer effect.

Although the abundance of *A. muciniphila*, a member of immunomodulatory bacteria, is related to immune response, some studies have reported that *A. muciniphila* in the feces exerted specific effects on colorectal cancer [52, 53]. However, *A. muciniphila* regulates immune response in tumor patients [19, 54–59]. *Akkermansiaceae* was significantly lower in the CRC group [60], and the addition of *A. muciniphila* significantly inhibited the tumorigenic effect of another flora [59]. It is also associated with favorable responses to antiprogrammed death protein 1 (PD-1) therapy [61]. In the present study, we study the changes in tumor volume and the expression of *A. muciniphila* in mice after oral administration of urolithin B by using two animal models, AOM/DSS chronic inflammation induced and APC^min/+^ adenoma colon cancer model. The tumor decreased significantly after the administration of urolithin B, followed by the downregulation of PD-L1 and increase of the expression of *A. muciniphila*. These results support the enhanced immune response of *A. muciniphila* in colon cancer.

Alloprevotella, which is correlated with higher levels of chromosomal aberrations [62], might contribute to the development of CRC [63]. In addition, the inhibition of PD-1 decreased the abundance of *Alloprevotella* [64]. Those reported results illustrated that *Alloprevotella* not only is related to the development of colon cancer but also participates in the immunotherapy of PD-1 of colon cancer. Our present results show that the combination of urolithin B with first-line therapeutic drugs can downregulate the expression of *Alloprevotella* and inhibit the expression of PD-L1. Notably, UB combined with anti-PD-1 antibody could inhibit the growth of colon cancer, which further provide a strategy for the treatment of immunotherapy for CRC treatments.

Overall, the prevention of colorectal carcinogenesis of urolithin B is attributed to the shape of gut microbial and tumor immune microenvironment. The anticancer concentration of urolithin B is consistent with the GI concentration of polyphenols, suggesting the protective effect of polyphenols against diseases.

## 5. Conclusion

**Figure 8:**
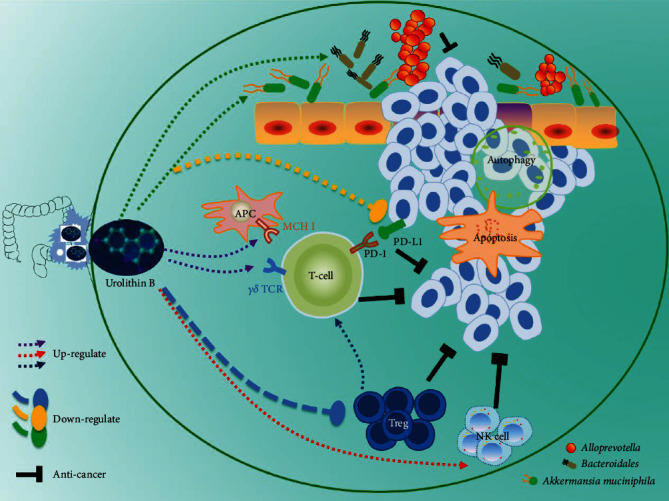
A schematic of the effect of urolithin B on colon cancer cells. Urolithin B, which is the final metabolite of polyphenols in the GI tract, inhibited colon cancer cell growth by remodeling gut microbiota and tumor immune microenvironment. The combination of UB with first-line therapeutic drugs improved the colon cancer immune microenvironment.

In summary, we showed that urolithin B, the final metabolite of polyphenols in the GI tract, prevented colorectal carcinogenesis by remodeling the gut microbial and tumor immune microenvironment (Figure 8). UB plays an antitumor role in five aspects. Firstly, UB kills tumor cells by enhancing the vitality of NK cells. Secondly, UB inhibits the activity of regulatory T cells to relieve and promote antitumor immunity. Thirdly, UB inhibits the expression of PD-L1, relieves immunosuppression, and promotes antitumor immunity. In addition, UB upregulates the expression of HLA-B and TCR, plays a role similar to the dendritic cell vaccine, enhances antigen presentation, and promotes antitumor immunity. Lastly, UB regulates the immunomodulatory flora in colon cancer and plays an antitumor role. Compared with the single use of capecitabine, the combination of urolithin B with first-line therapeutic drugs showed better body conditions and increased antitumor effects on colorectal carcinogenesis mice, by shaping gut microbia and providing a strategy for the treatment of immunotherapy for CRC treatments. UB combined with anti-PD-1 antibody could inhibit the growth of colon cancer. Urolithin B may thus contribute to anticancer treatments and provide a high immune response environment for CRC patients' further immunotherapy.

## Data Availability

Data generated during the processing or analysis that support the findings of this study are available from the author (lixue_wang@ccmu.edu.cn) on reasonable request.
